# Medication adherence in geriatric patients attending medical outpatient department

**DOI:** 10.4102/safp.v66i1.6011

**Published:** 2024-10-25

**Authors:** Oluremi A. Odubanjo, Brent Tipping, Lara S. Greenstein

**Affiliations:** 1 Department of Internal Medicine, Faculty of Health Sciences, University of the Witwatersrand, Johannesburg, South Africa; 2 Division of Geriatric Medicine, Helen Joseph Hospital, Johannesburg, South Africa; 3 Division of Geriatric Medicine, Wits Donald Gordon Medical Centre, Johannesburg, South Africa

**Keywords:** geriatric population, adherence, polypharmacy, medication, barriers, challenges

## Abstract

**Background:**

Adherence to medication represents a challenge in managing chronic conditions in the geriatric population. This study assessed adherence rates and factors affecting adherence of geriatric patients attending the Helen Joseph Hospital outpatient department.

**Methods:**

This was a prospective cross-sectional study of 130 patients aged 65 years and older, with at least two chronic conditions. Participants were administered a survey incorporating the Medication Adherence Rating Scale and the Adherence Barrier Questionnaire to identify medication adherence and patient-specific barriers to adherence, respectively. These instruments are reliable and valid.

**Results:**

Descriptive statistics and logistic regression were used for analysis. Most patients were female (63%) with a mean age of 72 (67–78) years. Common comorbidities included type 2 diabetes mellitus (63%), hypertension (98%), dyslipidaemia (92%) and congestive cardiac failure (38%). Polypharmacy was prevalent, affecting 53% of the participants. Despite 96% of participants being adherent, all had at least one barrier to adherence, with the majority (65%) having more than one barrier. The main barriers were forgetfulness (59%), fear of side effects (39%), problems with taking the medications (26%) and believing medications are poisonous (22%). Although most participants accessed the pharmacy easily, only 83% reported consistent medication availability and 11% could not afford to collect their medication.

**Conclusion:**

Polypharmacy is common in the population. Despite high adherence rates, barriers such as believing medications are poisonous remain significant. A good patient–doctor relationship improves adherence.

**Contribution:**

Understanding the barriers to adherence in older adults with polypharmacy and multimorbidity can assist practitioners improve patient care.

## Introduction

Ageing is an inevitable phenomenon and can be accompanied by declining health with consequent accumulation of chronic illnesses including cardiometabolic diseases, neurocognitive disorders and musculoskeletal conditions. Having multiple chronic conditions can culminate in a high burden of chronic medication use. Adherence to this medication can pose challenges. In South Africa, the number of older persons aged 60 years and above is estimated to be 5.6 million, 9.2% of the entire population.^1^ Although 65 years is the definition of geriatric worldwide, in South Africa and the rest of Africa, the World Health Organisation uses the definition of 60 years as an older person.^1^

Medication adherence is defined as the extent to which patient behaviours in taking medication and executing dietary and lifestyle changes align with recommendations from healthcare providers.^2^

Barriers to medication can be divided into categories comprising patient factors, medical factors, healthcare provider factors, healthcare systems factors and socio-economic factors.^3,4,5^ Challenges to adherence are a hindrance in the treatment of chronic illnesses in the older population. Non-adherence can be either intentional or non-intentional. Intentional non-adherence refers to the patient taking medication differently from the prescriber’s recommendations. Unintentional non-adherence refers to the patient wanting to take medication as prescribed but being unable to do so because of factors such as poor resources, a lack of understanding, forgetfulness, concerns about effectiveness, side effects and physical barriers such as poor dexterity and swallowing difficulties.^3,6^

Various subjective tools using questionnaires and scales are available to assist in the measurement of medication adherence. The Medication Adherence Rating Scale (MARS) is used to assess adherence based on three dimensions: medication adherence behaviour, attitude towards taking medication and attitudes to the negative side effects of prescribed medications.^7^ The higher the score, the better the adherence. The Adherence Barrier Questionnaire (ABQ) is a practicable, reliable and validated instrument for identifying patient-specific barriers to medication-related adherence. It is a 15-item scale comprising the barriers assessing intentional adherence, medication and healthcare system-related adherence and unintentional adherence.^8^

Polypharmacy can either be defined in terms of being prescribed more medication than necessary or in terms of the number of medications taken, usually more than five. These medications include prescribed medication, over-the-counter medication and nutraceuticals.^9^ The prevalence of polypharmacy in a South African hospital in the free state was very high at 84.3%.^10^ Apart from the potential of non-adherence, polypharmacy increases the risk of drug interactions and adverse drug reactions.^11^ Excessive polypharmacy is defined as the use of 10 or more medications, the consequences of which may lead to poor health outcomes.^12^ Appropriate pharmacy occurs when multiple medications are used in such a way that each one has a clear indication with minimum risk of harm.^13^ The hallmark of appropriate pharmacy is rational prescribing whereas polypharmacy can be harmful when not properly managed. With polypharmacy, the pill burden is often high and can lead to poor adherence.^14^ Inappropriate pharmacy pill burden is minimised by optimising treatment plans.

Multimorbidity refers to the presence of two or more chronic conditions, and together with polypharmacy, is one of the geriatric syndromes.^15,16^ Multimorbidity and polypharmacy are closely related as multimorbidity often requires the use of numerous medications. Both polypharmacy and multimorbidity may impact a number of other geriatric syndromes such as frailty.^17^

In the paucity of published studies on adherence to medication in geriatric patients at medical outpatient departments (MOPD) in South Africa, we aimed to investigate medication adherence and the barriers to this adherence in geriatric patients at the Helen Joseph Hospital MOPD.

## Research methods and design

### Study design, setting and period

This was a prospective cross-sectional descriptive study undertaken at the Helen Joseph Hospital MOPD. Helen Joseph Hospital is situated in Auckland Park, Johannesburg and serves a population of approximately 1 million people. Patients are referred to MOPD from in-ward discharges, district-level hospitals, the in-hospital polyclinic, the emergency department and surrounding primary healthcare clinics and smaller district clinics. Data collection was over 7 months from April 2021 to October 2021.

### Study population, sample size and sampling methods

All geriatric patients with at least two chronic conditions and a follow-up period of at least 3 months were eligible for this study. Geriatric was defined as age 65 years and older. Chronic conditions included any long-term medical illness, which had been present for at least 3 months. Between 1280 and 1440 patients are seen at MOPD per month of which approximately 250 are 65 years and above. The sample size was calculated to be 130 patients. This is based on a 95% confidence interval with a 5% margin of error. All potential participants who attended Friday MOPD were identified while waiting in the queue and informed consent was obtained from those who agreed and who were eligible based on the inclusion and exclusion criteria. Verbal consent was obtained from the patients who then signed a consent form in English with a nursing sister translating the form into Zulu when necessary. A total of 157 patients were eligible and 130 agreed to participate. Those who agreed were seen in a private consulting room in the morning prior to 09:00 when MOPD started so as not to impact their appointments. Each patient at MOPD receives a number ensuring that participating in the study did not result in losing their place in the queue. Questionnaires were administered in English and the same nursing sister assisted with translation into Zulu when required to ensure reliable results.

### Data collection

The study survey was divided into four sections. Clinico-demographic data included age, marital status, chronic illness diagnoses and a list of medications including the drug name, duration of treatment and frequency of dosing.

The second section comprised the validated ABQ^8^ which is a 15-item questionnaire with a Likert scale consisting of ‘strongly agree’, ‘generally agree’, ‘generally disagree’, ‘strongly disagree’, ‘not applicable’ and ‘do not know’. These answers are given values from 1 to 4 with a score of 2 or more indicating that the patient has a higher perception of that item being a barrier.

The third section is the MARS^7^, a self-report 10-item instrument comprising a yes/no response to questions asked. The first four questions address medication adherence behaviour, the following four questions address attitudes towards taking medication and the final two questions address side effects and attitudes towards psychotropic medication. Non-adherence consistent responses were coded as 0, adherence consistent responses were coded as 1. A ‘no’ response to questions 1–6 and 9–10, and ‘yes’ response to questions 7 and 8 were indicative of adherence. A score of less than 5 was considered low adherence and a score of 5 or more was considered adequate adherence.^18^ Permission to use the scale was obtained from the original author.

The fourth section of the survey addressed other factors affecting adherence that were not addressed by the two validated questionnaires. These included patient factors (hearing problems, visual disturbances, swallowing problems and alcohol use), medication-associated factors (doses per day, number of medications (pill burden), how often medication is forgotten and knowledge of the reason for medication use), healthcare provider factors, healthcare systems factors (access and availability) and socio-economic factors (level of education and access to transport). Poor socio-economic status was determined by either a poor level of education or the reliance on a grant as the only form of income. These questions were structured as yes/no questions.

### Statistical methods

Data were captured on Research Electronic Data Capture (REDCap) and statistical analysis was carried out using STATA 15 (STATA Corp MP version 16). Descriptive statistics were used in the data analysis. For continuous variables that did not follow a normal distribution, the median and interquartile range (IQR) were reported. The Chi-square test was used to compare categorical variables. Mean and standard deviations were used to summarise normally distributed continuous data. The student *t*-test was used to compare the means from the normally distributed continuous variables and the Wilcoxon rank sum test was used for comparing medians from nonparametric variables. Univariate and multivariate logistic regression was used to determine factors impacting adherence. These predictors were presented using odd ratios with a confidence interval of 95%. Variables that achieved < 0.2 in the univariate analysis were included in the multivariate analysis. A *p*-value ≤ 0.05 was considered statistically significant.

### Ethical considerations

Permission to conduct the study was approved by the Human Research Ethics Committee (HREC) of the University of the Witwatersrand (clearance certificate number M211169). Study participants gave informed consent before they were enrolled and all identifying data were removed to allow for anonymity. Patient participation was voluntary and no patient was denied services if they refused participation. No incentive or reimbursement was offered to those who participated. To ensure patient confidentiality, no patient identifiers were used when capturing the data. Digital data were stored on a password-protected computer accessible to only the researchers and will be stored for 5 years after study completion.

## Results

A total of 157 patients were approached for this study and 27 were excluded as they did not provide consent. A total of 130 patients participated in the study.

### Demographic and clinical details

The demographic and clinical characteristics of the geriatric patients attending Helen Joseph Hospital MOPDs are shown in Table 1. Most patients were female (63%), with a median (IQR) age of 72 (67–78) years. Just over 40% of patients obtained a primary school level of education and just over half (51%) of the participants were married. Very few of the patients (6.92%) were smokers. The majority of participants (72.31%) reported having visual problems and almost one-third (34.62%) reported hearing problems.

**TABLE 1:** Demographic and clinical characteristics of geriatric patients attending Helen Joseph Hospital medical outpatient department stratified according to the adherence by the Medication Adherence Rating Scale.

Variable	All participants (total: *N* = 130, 100%)				Non-adherent according to MARS (*n* = 5, 3.85%)				Adherent according to MARS (*n* = 125, 96.15%)				*P*
	Median	IQR	*n*	%	Median	IQR	*n*	%	Median	IQR	*n*	%	
Age (years)	72	67–78	-	-	72	68–81	-	-	72	67–78	-	-	0.88
Female gender	-	-	82	63.0	-	-	2	40	-	-	80	64.00	0.27
**Marital status**	-	-	-	-	-	-	-	-	-	-	-	-	0.37
Single or unmarried	-	-	25	19.2	-	-	0	0	-	-	25	20.00	-
Married	-	-	51	39.2	-	-	2	40	-	-	49	39.20	-
Divorced	-	-	16	12.3	-	-	0	0	-	-	16	12.80	-
Widow/widower	-	-	33	25.4	-	-	3	60	-	-	30	24.00	-
**Level of education**	-	-	-	-	-	-	-	-	-	-	-	-	0.49
Primary	-	-	53	40.8	-	-	3	60	-	-	50	40.00	-
Secondary	-	-	60	46.2	-	-	1	20	-	-	59	47.20	-
Tertiary/Higher degree	-	-	17	13.1	-	-	1	20	-	-	16	13.08	-
Hearing problem	-	-	45	34.6	-	-	3	60	-	-	42	33.60	0.47
Visual problems	-	-	94	72.3	-	-	5	100	-	-	89	71.20	0.37
Smoker	-	-	9	6.9	-	-	0	0	-	-	9	7.20	0.77
Diagnosis	-	-	-	-	-	-	-	-	-	-	-	-	-
Diabetes mellitus type 2	-	-	48	36.9	-	-	4	80	-	-	44	35.20	**0.04**
Hypertension	-	-	127	97.7	-	-	5	100	-	-	122	96.60	0.73
Dyslipidaemia	-	-	119	91.5	-	-	4	80	-	-	115	92.00	0.34
Ischaemic heart disease	-	-	24	18.5	-	-	1	20	-	-	23	18.40	0.93
Chronic kidney disease	-	-	13	10.0	-	-	2	40	-	-	11	8.80	**0.02**
Congestive cardiac failure	-	-	41	31.5	-	-	1	20	-	-	40	32.00	0.57
Chronic obstructive pulmonary disease	-	-	16	12.3	-	-	0	0	-	-	16	12.80	0.39
Asthma	-	-	9	6.9	-	-	0	0	-	-	9	7.20	0.53
Thyroid disorders	-	-	14	10.8	-	-	0	0	-	-	14	11.20	0.43

Note: Data in bold is statistically significant.

MARS, Medication Adherence Rating Scale; IQR, interquartile range.

The commonest comorbidities included type 2 diabetes mellitus (36.92%), hypertension (97.69%), dyslipidaemia (91.54%) and congestive cardiac failure (37.54%).

The most commonly prescribed medications included antihypertensive agents, statin therapy, anti-diabetic agents and aspirin.

A statistically significantly higher proportion of patients with type 2 diabetes mellitus (*p* = 0.042) and chronic kidney disease (*p* = 0.025) were non-adherent to their medication. The level of education did not correlate with adherence.

### Adherence to medication

**FIGURE 1:**
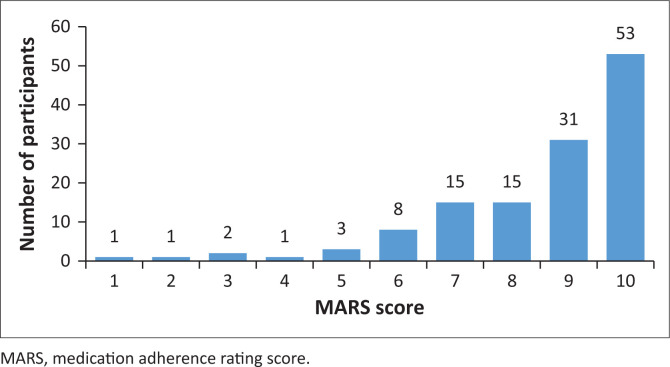
The medication adherence rating score.

As shown in Figure 1, 96% of participants were adherent to their medication.

### Barriers to adherence

**FIGURE 2:**
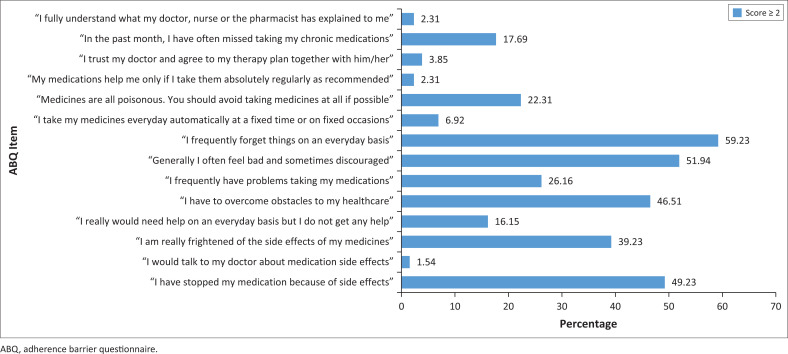
Proportion of patients affected by barriers to adherence using the barriers to adherence questionnaire.

All participants had at least one barrier to adherence as defined by the ABQ, with the majority of participants (65.38%) having between two and five barriers. The most common barriers to adherence included forgetfulness (59.23%), being afraid of side effects (39.23%), problems with taking the medication (26.15%), believing medications are poisonous and should be avoided (22.31%) and missing doses (17.69%) (see Figure 2).

Barriers to adherence that are not included in the validated ABQ are shown in Table 2. These barriers are divided into patient factors, medication factors, healthcare provider factors, health systems factors and socio-economic factors. Only 5% of participants forgot to take their medication more than five times in the preceding month.

**TABLE 2:** Factors affecting adherence among geriatrics patients attending the Helen Joseph Hospital medical outpatient department.

Variable	All participants (total: *N* = 130, 100%)				Non-adherent according to MARS (*n* = 5, 3.85%)				Adherent according to MARS (*n* = 125, 96.15%)				*P*
	*n*	%	Median	IQR	*n*	%	Median	IQR	*n*	%	Median	IQR	
**Medication factors**	-	-	-	-	-	-	-	-	-	-	-	-	-
**Medication doses per day**	-	-	-	-	-	-	-	-	-	-	-	-	0.1100
Once	26	20.0	-	-	0	0	-	-	26	20.8	-	-	-
Twice	76	58.5	-	-	2	40	-	-	74	59.2	-	-	-
Thrice	25	19.2	-	-	3	60	-	-	22	17.6	-	-	-
Quadruple	3	2.3	-	-	0	0	-	-	3	2.4	-	-	-
**Number of pills taken each day**	-	-	-	-	-	-	-	-	-	-	-	-	0.8800
0–5	61	46.9	-	-	2	40	-	-	59	47.2	-	-	-
6–10	66	50.8	-	-	3	60	-	-	63	50.4	-	-	-
11+	3	2.3	-	-	0	0	-	-	3	2.4	-	-	-
Average number of medications	-	-	6	5–7	-	-	6	5–7	-	-	6	5–7	0.9200
**Number of times medication was omitted in the preceding month**	-	-	-	-	-	-	-	-	-	-	-	-	**0.0079**
0–5	123	94.6	-	-	4	80	-	-	119	95.2	-	-	-
6–10	4	3.1	-	-	1	20	-	-	3	2.4	-	-	-
11–15	3	2.3	-	-	0	0	-	-	3	2.4	-	-	-
16–20	-	-	-	-	-	-	-	-	-	-	-	-	-
Good knowledge of what medication is for	128	98.5	-	-	5	100	-	-	123	98.5	-	-	0.7800
Health provider factors	-	-	-	-	-	-	-	-	-	-	-	-	-
Good relationship with the doctor	123	94.6	-	-	3	60	-	-	120	96.0	-	-	**< 0.0010**
**Healthcare system factors**	-	-	-	-	-	-	-	-	-	-	-	-	-
Easy access to the pharmacy	123	94.6	-	-	4	80	-	-	119	95.2	-	-	0.1400
Availability of medication	108	83.1	-	-	4	80	-	-	104	83.2	-	-	0.9500
Affordability of medications	115	88.5	-	-	3	60	-	-	112	88.6	-	-	0.0900

Note: Data in bold is statistically significant.

MARS, medication adherence rating score; IQR, interquartile range.

With respect to factors that promote adherence, Table 2 shows that 98% of participants knew what their medication was used for and 95% had a good relationship with their healthcare providers. Most participants, 95%, found it easy to access the pharmacy yet only 83% of participants found their medication was always available and 11% of participants could not afford to collect their medication every month. The majority of participants, 53%, had polypharmacy, using more than five medications per day while 2% had excessive polypharmacy with the use of 10 or more medications per day. The dosing of medication was most often bi-daily (58%) with only 2% of participants needing to take medication more than three times per day. In keeping with our high rate of adherence, only 5% of patients omitted taking their pills more than five times in a month.

Table 3 shows some of the independent factors associated with adherence in our study participants. Having a good relationship with the healthcare provider and the affordability of medication tended towards statistical significance.

**TABLE 3:** Univariate and multivariable logistic regression factors affecting medication non-adherence.

Variable	Univariate		*p*	Multivariate		*p*
	OR	95% CI		OR	95% CI	
Age	0.99	0.88–1.13	0.907	-	-	-
Female gender	2.67	0.43–16.56	0.292	-	-	-
Married	2.45	0.39–15.52	0.341	8.58	0.21–358.17	0.259
Secondary level of education	3.54	0.36–35.11	0.280	-	-	-
Tertiary/higher degree	0.96	0.09–9.89	0.973	-	-	-
Hearing problem	0.34	0.05–2.12	0.249	-	-	-
Twice daily dosing	5.05	0.79–32.13	0.087	9.03	0.46–178.43	**0.148**
Pill burden: 6–15	0.77	0.12–4.80	0.783	-	-	-
Forgetting to take pills more than five times a month	0.10	0.01–1.20	0.069	-	-	-
Good relationship with the doctor	16	2.16–118.27	0.007	-	-	-
Easy access to pharmacy	4.96	0.48–51.46	0.180	5.00	0.10–253.23	0.421
Availability of medication	1.3	0.14–12.25	0.819	-	-	-
Affordability of medication	6.22	0.94–41.01	0.057	1.88	0.05–67.81	0.731

Note: Data in bold is statistically significant.

OR, odds ratio; CI, confidence intervals.

## Discussion

According to the World Health Organization, adherence rates in developing countries are likely worse than the 50% adherence rates seen in developed countries.^19^ This is important because non-communicable diseases contribute a large proportion to the global disease burden.^19^ Older people are more likely to have multiple chronic conditions, a higher rate of polypharmacy and a higher prevalence of non-adherence because of the high pill burden, adverse drug reactions and drug–drug interactions. Non-adherence itself is associated with adverse health outcomes such as hospitalisation, emergency department visits and mortality.^20^

The demographic breakdown of our study showed that the majority of participants were female, which may give some insight into the health-seeking behaviour of patients in our hospital’s drainage area. Just over 40% of participants had only a primary level of education, which may reflect the poorer socio-economic status of our patient population; however, this did not have an impact on adherence in our study. Previous studies showed that marital status influenced medication adherence;^21^ however, this was not the case in our study.

In our study, the self-reported adherence rate was very high at 95%. This contrasts with other studies conducted in Africa. A study conducted in North-West Ethiopia showed that 25.4% of participants of all ages had low adherence. Unlike our study group, this group of patients all had type 2 diabetes. Our results showed that patients living with type 2 diabetes were less compliant than their non-diabetic counterparts.^22^ In a large Chinese study, the prevalence of non-adherence in geriatric outpatients visiting tertiary centres was as high as 31.8%.^23^ In India, the prevalence of poor adherence was only 2%, which is very similar to our study.^23^ The differences in the prevalence rates in all of these studies could be because of the variety of different tools used to assess adherence and differences in the population groups and the study settings. Our hospital is a tertiary institution and cannot be directly compared to non-tertiary level hospitals. Almost 95% of our participants acknowledged that the medication helped only if they were taken regularly as recommended. Most of our participants took their medicines regularly at a fixed time.

Factors that influence adherence can be divided into those that are modifiable and those that are non-modifiable. Potentially modifiable barriers include disease-related knowledge, health literacy, social support and polypharmacy.^24,25^ These are the barriers that should be given priority as this is where meaningful change can be achieved.

Despite the high rate of adherence, 96% of the participants in our study had at least one barrier to adherence. These barriers included patient factors, medication factors, healthcare provider factors, health systems factors and socio-economic factors. Transport issues and stock availability were some of the issues highlighted in this study. A qualitative study conducted in Iran showed a number of themes relating to poor adherence.^26^ These included medication errors, forgetfulness, cultural factors, socio-economic factors and fear of complications.^26^ A scoping review that included barriers to adherence in low- to middle-income countries such as ours showed that lack of knowledge about prescribed medication, negative attitudes and negative beliefs influenced adherence.^27^ The use of traditional medicines is common in South Africa, and this has been associated with poor adherence in other studies, particularly in people living with diabetes.^22^ Reasons for this may include patients’ attitudes and the preference to consult with traditional healers but the relationship is not completely understood. This does however highlight the importance of asking our patients whether they use traditional medications, and if so, how they use them.^22,28^

Medication-specific barriers are a common theme among studies looking at non-adherence. In our study, 39% of participants were worried about the side effects of their medication and 49.23% had stopped at least one medication because of patients experiencing side effects. Worryingly, 22% of participants believe medications are poisonous and should be avoided if possible. We extrapolate that this may be because of patients experiencing side effects or may be because of cultural factors. Attitudes to different medications may be influenced by socio-demographic and cultural factors.^29,30^ The use of traditional medicine was shown to be independently associated with poor adherence in a study focussing specifically on sub-Saharan countries.^31^ Hypertensive patients in America reported medication side effects, pill size and difficulty swallowing pills as some of their barriers to adherence.^32^ Some studies have shown that complicated regimes are a barrier to adherence^33^ and this is of particular importance in the geriatric population where forgetfulness is a major contributor to non-adherence. In fact, our study showed that 59% of patients had forgotten to take their prescribed medication at least once in the preceding month.

Certain healthcare-related factors such as the availability and accessibility of medications were not statistically significant in this study, but they still deserve a mention because stock shortages are relatively common in our setting and the financial burden of transportation impacts adherence.

Our study showed that having a good relationship with the healthcare provider meant adherence was more likely. Ninety-eight per cent of our cohort fully trusted their health care practitioner and understood what had been explained to them. In our study, most patients knew what their medication was for and trusted their healthcare provider. Other studies corroborate this finding – in a primary health care setting, adherence was improved by good communication with the treating doctor, social support and knowledge about the disease and treatment thereof.^32^

One of the most common geriatric syndromes is multimorbidity or the presence of two or more chronic health conditions. In our study, the most common diseases seen included type 2 diabetes mellitus, hypertension, dyslipidaemia and congestive cardiac failure. These conditions seem to be the most common conditions seen in other studies exploring adherence in older patients.^34^ In our hospital setting, organ-specific diseases are often seen at dedicated sub-specialist clinics and not at MOPD. Certain comorbidities may be underrepresented in our study.

Our study showed a 53% prevalence of polypharmacy, a lot higher than the prevalence in a study conducted in a dedicated geriatric centre in Nigeria.^35^ In that study, polypharmacy was associated with factors leading to non-adherence such as visiting many different clinics and intentionally missing medication doses because of high pill burden. In Durban, a study conducted at a regional hospital revealed that the average number of prescribed medicines was 12.^11^ In our study, only 2% of participants were prescribed 10 medications or more. Moreover, in the Durban study, the highest prevalence of polypharmacy was found in the 60–65-year age group. In an American study, the average number of medications was 5.7 prescription drugs.^33^ A recent systematic review published in the Lancet showed the prevalence of polypharmacy ranged from 2.6% to 86.6%. Although our study was not primarily focussed on polypharmacy, the high rate of polypharmacy may increase adverse drug reactions and drug–drug interactions which in turn lead to further polypharmacy and the creation of prescription cascades.^36,37^ In older adults with multimorbidity and polypharmacy, medication side effects can be mistaken for new conditions. This leads to further prescribing which can cause poor outcomes and further drug-drug interactions.^36^

### Strengths and limitations

The biggest strength of this study is that it focusses on older patients, the group that is often prescribed the most medication. The study looks at a number of factors affecting adherence using validated questionnaires. This study does however have multiple limitations including a small sample size and being conducted in a single centre. In our study, we asked only about prescription medications and not over-the-counter and traditional medications. This study was also undertaken in an urban setting at a tertiary-level hospital so the results may not be generalisable to other centres or rural settings. This study did not address geriatric syndromes such as frailty and dementia which may have an impact on adherence. The study relied on self-reporting by patients with no corroborative additional objective measure of assessing adherence such as the medication possession ratio (MPR) and the proportion of days covered (PDC).^38^

### Future recommendations

Ensuring a strong commitment from all stakeholders including the National Department of Health, the Pharmaceutical Society of South Africa, healthcare professionals, health planners, policymakers and researchers is vital in improving barriers to medication adherence. Patients themselves have a right to be educated on what medications they are receiving, what their effects are, the common side effects and the duration of use. They should also have ready access to medications and healthcare system-related factors that impact adherence need to be mitigated. The patients themselves have a responsibility to take their medications as prescribed.

Based on our findings, we can recommend that practitioners establish good relationships and connect with their patients by listening to them and addressing their concerns. This includes explaining to them exactly what their chronic conditions are, how the medications work and what side effects to expect. Healthcare provider education is required to assist with this. Adequate stock levels in pharmacies must be ensured as 17% of patients reported stock unavailability. The ability of all patients to access pharmacy services is paramount.

Finding solutions to help older persons remember to take their medication is needed – this may be arranged by simplifying dosing regimens, asking family members for help, using pill boxes and the use of apps on cellular phones. A case may be made for the need for memory clinics to be incorporated into hospital services.

Patients need to be informed about the importance of taking their medications and the risk-benefit ratio to reduce their perception of medications being poisonous.

## Conclusion

Medication adherence is of paramount importance in the management of chronic conditions. Multimorbidity and polypharmacy are common challenges, and as such, adherence in this population can be problematic. Our study showed a high rate of adherence, but despite this, the majority of patients had at least one barrier to medication adherence. The main factor that improved adherence was a good patient–doctor relationship. Perceptions of medications being poisonous need to be addressed in our patient population.
